# Clinical Characteristics and Risk Factors of Recurrent Mooren's Ulcer

**DOI:** 10.1155/2017/8978527

**Published:** 2017-06-27

**Authors:** Lixia Yang, Juan Xiao, Jiawei Wang, Han Zhang

**Affiliations:** ^1^Department of Ophthalmology, The Second Hospital of Shandong University, Jinan, Shandong, China; ^2^Center of Evidence-Based Medicine, The Second Hospital of Shandong University, Jinan, Shandong, China

## Abstract

**Purpose:**

To investigate the clinical characteristics of Mooren's ulcer in East China and to identify the potential risk factors that affect the recurrence of Mooren's ulcer.

**Methods:**

We reviewed the medical records of 95 patients (100 eyes) diagnosed with Mooren's ulcer from May 2005 to December 2014. The patients were classified into recurrent and nonrecurrent groups and followed up for 18 months. The difference between two groups was estimated. The patients in the recurrent group were subdivided according to the history of corneal infection and corneal perforation, respectively. The recurrent time in the subgroups was analyzed.

**Results:**

Patients in the recurrent group were more likely to have a history of corneal infection and corneal perforation than that in the nonrecurrent groups. In patients with recurrent Mooren's ulcer, the median time to first recurrence was 130 days in the infection group, 480 days in noninfection group, and 195 days in the perforation group versus 480 days in nonperforation group.

**Conclusion:**

Corneal infection and corneal perforation were associated with early recurrence of Mooren's ulcer. The tailored follow-up schedule should be used for patients with corneal infection and corneal perforation due to the high risk of recurrence.

## 1. Introduction

Mooren's ulcer is a painful, relentless, chronic ulcerative keratitis that begins peripherally and progresses circumferentially and centrally [1]. The disease was named by Mooren who first clearly described this insidious corneal problem and defined it as a clinical entity [1]. It can be either unilateral or bilateral [2]. The scleras of patients characteristically remain quiescent. Although organ-specific autoimmunity is the most accepted theory [3], the etiopathogenesis of the disease remains unclear.

The incidence, clinical characteristics, and severity of Mooren's ulcer widely vary geographically and racially [4]. Epidemiological studies suggest that the disease is rare in the northern hemisphere but common in southern and central Africa, China, and India [5].

The essential aim of the treatment is to promote the epithelialization, to control the inflammation, and to prevent the progression. Treatment options include medications and surgery. However, there is not enough evidence to show which one is the most effective. The incidence of recurrent Mooren's ulcer is high. Chen et al. [6] reported that 25.6% of postoperative patients experienced at least one recurrence and even higher in those with malignant ulcer. The management of recurrence is still considered to be a great challenge for many ophthalmologists. A great number of recurrent patients eventually suffer from poor eyesight. However, limited literature was available on the risk factors of recurrent Mooren's ulcer.

In our study, we examined the clinical characteristics of recurrent Mooren's ulcer in East China and investigated the potential risk factors associated with the recurrence of this disease. Our findings may provide some evidence to help ophthalmologists select the most appropriate treatment and decrease the risk of recurrence.

## 2. Patients and Methods

This was a retrospective study. We reviewed medical records of 100 eyes from 95 patients. Ninety patients had unilateral lesions, and five patients had bilateral lesions. All patients were from East China and were admitted to Shandong Eye Hospital of Shandong Eye Institute between May 2005 and December 2014 with a diagnosis of Mooren's ulcer. The diagnosis of Mooren's ulcer had been established by the typical clinical features: a painful, crescent-shaped, peripheral corneal ulcer which starts at the limbus with a gray overhanging infiltrated margin and no sclera involved. Corneal smear and corneal culture were used to rule out the corneal infection. Erythrocyte sedimentation rate, antistreptolysin O, and rheumatoid factor were tested to exclude systemic diseases such as rheumatoid arthritis, systemic lupus erythematosus, and Wegener's granulomatosis, which could cause peripheral corneal ulcer (PUK).

The first investigation day for each patient was the day the patient was present in our hospital. Each patient received slit-lamp microscopy at 1 week, 1 month, 3 months, 6 months, 12 months, and 18 months after the first visit.

We divided the patients into two groups: recurrent group and nonrecurrent group. The patients in the recurrent group were those who visited our clinic for the second time because of recurrent Mooren's ulcer during the study period.

There were nine patients to whom recurrence occurred repeatedly (the frequency of recurrence > 3) over the study period between May 2005 and December 2014, and their clinical characteristics were assessed and displayed.

We collected the demographic information including gender, age, occupation, and urbanicity (urban or rural). The clinical characteristics we examined included laterality of the eye affected by the disease. Ocular history (including ocular surgery, corneal infection, and ocular trauma), presence of corneal perforation, corneal neovascularization, corneal epithelial defect, corneal loose suture, clock hours of ulcer involvement (≤3; >3 and ≤6; >6 and ≤9; >9 and ≤12), surgery methods, initial visual acuity, and final visual acuity. All the above factors we collected were compared between the two groups to identify the risk factors related to recurrence of Mooren's ulcer.

### 2.1. Medical Therapy

The patients took antibacterial eye drops, 0.1% dexamethasone eye drops, tobramycin, and dexamethasone ointment once daily at night. For the aggressive cases, 1% cyclosporine A or 0.1% tacrolimus eye drops might be used 4 times daily. The use of topical steroids was tapered based on the patient's response to the treatment. Systemic immunosuppression was recommended only if patients did not respond to the local therapy.

### 2.2. Surgical Treatment

Surgical treatment was performed in the cases with actual or impending perforation and in the cases with noneffective medical treatment. Amniotic membrane transplantation (AMT) or conjunctival flap (CF) was performed when the ulcer was shallow, with a depth of less than 50% of the corneal stroma. If the depth of ulcer was more than 50% of the corneal stroma, the partial lamellar keratoplasty (LKP) or total lamellar keratoplasty (LKP) was applied.

### 2.3. Statistical Methods

The data were statistically analyzed using SPSS software version 20.0 (SPSS Inc., Chicago, IL, USA). Numerical variables which met the normal distribution were showed in “mean ± standard deviation” (age), and the two groups were compared by the independent two-sample *t*-test. Numerical variables not normally distributed were showed in “median (P_25,_ P_75_)” (inpatient days), and the two groups were compared by rank sum test. For the categorical variable, chi-square was used to test the difference of the proportions between two groups. For the patients in the recurrent group, Log rank test was used to compare the recurrence time between the subgroups with and without the history of corneal infection and corneal perforation. The level of significance was set at *p* < 0.05.

The Institutional Review Board (IRB) of Shandong Eye Hospital of Shandong Eye Institute approved our use of medical records. All procedures complied with the Declaration of Helsinki for research involving human subjects. All patients signed an informed consent approved by the IRB.

## 3. Results

### 3.1. The Demographics of Patients and Clinical Characteristics of Affected Eyes

To identify the factor(s) that affected recurrence, we compared the demographic and clinical characteristics between patients with and without recurrence (Tables 1 and 2).

In Table 1, a total of 95 patients were classified into the recurrent group (*N* = 42, 44.2%) or the nonrecurrent group (*N* = 53, 55.8%). There was no statistical difference in gender, age, urbanicity, and laterality of eyes between the recurrent group and the nonrecurrent group. The mean age at the time of diagnosis was 50 ± 14 years (18–74 years).

In Table 2, among 100 cases with recurrent Mooren's ulcer, 44 (44%) were in the right eye and 56 (56%) were in the left eye. Nineteen eyes (19%) had received ocular surgery including 8 eyes undergoing cataract surgery with clear corneal incision, 7 eyes undergoing pterygium excision, and 4 eyes undergoing glaucoma surgery. Seven eyes (7.3%) had a history of ocular trauma including 4 eyes scratched by vegetative branches and 3 eyes with ocular blunt injury. Of the 19 patients with a history of corneal infection, 11 had a history of corneal bacterial infection: 5 were *Staphylococcus aureus*, 2 had *Streptococcus pneumoniae*, and 4 had *Staphylococcus epidermidis*. Five patients had a history of herpes simplex virus infection, and three patients had a history of Fusarium infection. We were only able to identify the corneal loose suture among those patients who received keratoplasty during the first hospitalization. In the recurrent group, there were 6 eyes (13%) with corneal loose suture and 39 (87%) without corneal loose suture. There were 37 (37%), 34 (34%), and 16 (16%) eyes in clock hours of ulcer involvement of ≤3; >3 and ≤6; and >6 and ≤9, respectively.

The history of corneal infection and the history of corneal perforation were both significantly different between the recurrent group and the nonrecurrent group (shown in Table 2, *p* = 0.019 and 0.002, resp.).

### 3.2. Clinical Treatment and Outcome of Affected Eyes

**Figure 1:**
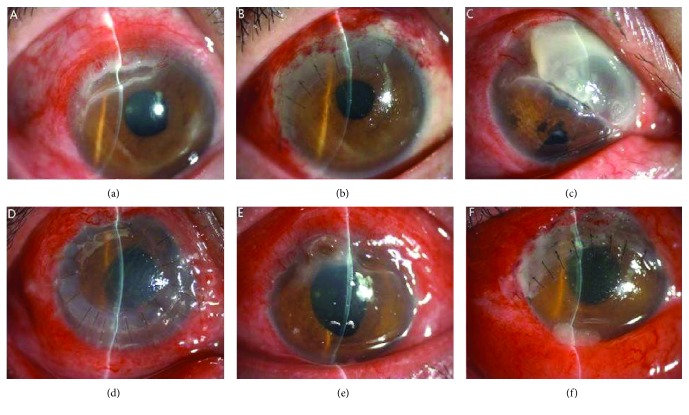
Slit lamp photograph of 3 patients with Mooren ulcer. (a) A 40-year-old man was first diagnosed with Mooren ulcer. At presentation, peripheral ulceration of the cornea was noted from the 9 o'clock to 12 o'clock position. (b) The image of the affected eye after receiving partial lamellar keratoplasty (LKP). In the following period, the patient did not have a recurrence. (c) A recurrent patient with Mooren ulcer presented with pain and decreased visual acuity. (d) The image of the affected eye after receiving total lamellar keratoplasty (LKP). (e) A patient with corneal perforation in 11 o'clock position presented in our hospital who was first diagnosed with Mooren ulcer. (f) The image of the affected eye after receiving corneal perforation repair with lamellar cornea and partial lamellar keratoplasty.

The clinical treatment and outcomes between patients with and without recurrence were also analyzed (Table 3). The results were displayed in Tables 3 and 4 and Figure 1.

For the initial treatment strategy, 20% of patients in the nonrecurrent group and 6.7% of patients in the recurrent group received medicine treatment (Table 3). No statistical difference was found in treatment methods, inpatient days, and visual acuities between the two groups (Tables 3 and 4).

Figure 1 shows the images of three typical eyes: one was in the recurrent group, one was in the recurrent group with corneal perforation, and one was in the nonrecurrent group.

The clinical characteristics of the patients with multiple recurrences are shown in Additional Table available online at https://doi.org/10.1155/2017/8978527. The patient's comprehensive medical history was evaluated, including clock hours of ulcer involvement, recurrence-free survival, signs of admission, manifestation of affected eyes, and surgery methods.

The initial manifestations of affected eyes were red, pain, and decreased eyesight in the patients with repeatedly recurrent Mooren's ulcer. The position of recurrence was almost the primarily affected position or corneal graft. Partial LKP or total LKP were the main treatment for these patients.

### 3.3. Survival Analysis Categorized by Corneal Infection and Corneal Perforation

**Figure 2:**
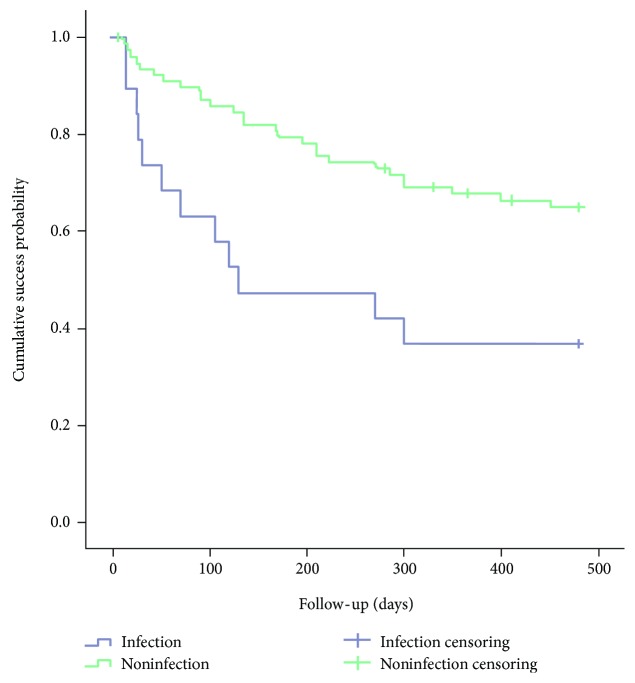
Comparison of survival curve between corneal infection group and noninfection group.

Chi-square analysis indicated that the history of corneal infection and the history of corneal perforation were likely related with the development of recurrent Mooren's ulcer. Based on this, we divided the eyes with Mooren's ulcer into two groups according to the history of corneal infection: 19 eyes with a history of corneal infection and 81 eyes without (Table 2). Significant statistical difference was found in the survival curves of the two groups (*p* = 0.005) (Figure 2). The median time interval from the initial diagnosis to first recurrence in the infection group was dramatically shorter than that in the noninfection group (130 days versus 480 days).

**Figure 3:**
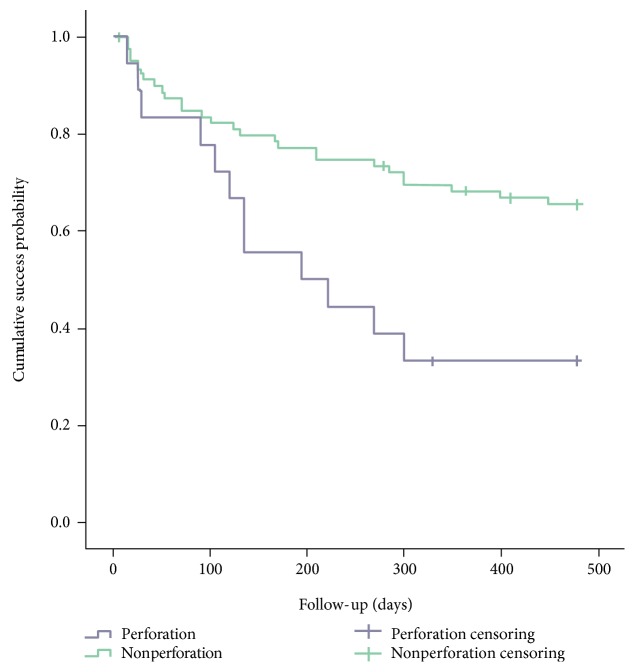
Comparison of survival curve between corneal perforation group and nonperforation group.

Next, we divided eyes with Mooren's ulcer into two groups according to the history of corneal perforation: 18 eyes with a history of corneal perforation and 82 eyes without (Table 2). Statistical significant difference was found in survival curves between the two groups (*p* = 0.007) (Figure 3). The median time interval from the initial diagnosis to first recurrence in the perforation group was dramatically shorter than that in the noninfection group (195 days versus 480 days).

## 4. Discussion

Mooren's ulcer is a relentless PUK characterized by stromal loss and absence of identifiable systemic disease [7, 8]. The prevalence of Mooren's ulcer and the blindness caused by the disease is unknown [4]. Recurrence of Mooren's ulcer is still a big issue in the management of Mooren's ulcer. The purpose of this study is to investigate risk factors for the recurrence of Mooren's ulcer and the clinical characteristics of ulcer recurrence and to identify proper treatment for reducing the risk of recurrence.

We examined the clinical characteristics of 100 eyes from 95 patients with Mooren's ulcer, who were admitted to the Shandong Eye Hospital over the past decade. In our study, the average age at onset of the disease was about 50 years old. The mean age of the recurrent group was 47 ± 14 years, and the mean age of the nonrecurrent group was 52 ± 14 years (*p* = 0.116). The majority of patients were men (1 : 0.56 or 61 versus 34). Chen et al. [6] found that among 550 patients with Mooren's ulcer from most regions of China, the average age of diagnosis was 48.4 years and the ratio of males to females was 1 : 0.74. In 1971, Wood and Kaufman [9] reported that Mooren's ulcer is more common in men, which is consistent with our findings. Our study estimated a recurrence rate of 44.2%, which was higher than the 25.6% recurrence rate reported by Chen et al. [6].

The rate of corneal perforation in our study was similar to the findings in the study by Zegans and Srinivasan (18% and 19%, resp.) [10]. Our findings were higher than those reported by Chen et al. (13.3%) [6] and lower than those reported by Kietzman (33.3%) [11], who conducted an observational study of 37 patients diagnosed with Mooren's ulcer in Nigeria in 1968.

The clinical characteristics of Mooren's ulcer vary greatly among the regions of the country. Age, gender, and race cannot be used as universal predictors of disease severity, process, or prognosis, especially in China and Asian India [3]. Previous studies supported that corneal trauma, surgery, and infection were risk factors for Mooren's ulcer [12–14]. Zegans and Srinivasan [10] found that a history of corneal trauma, surgery, or infection was reported in 68% of 21 patients with Mooren's ulcer from South India. Srinivasan et al. [3] conducted a study in South India and reported that in patients with Mooren's ulcer, 26% of cases had ocular trauma and 37% had previous ocular surgery, which indicated that prior disruption of corneal tissue might be a factor in inciting various inflammatory responses to finally induce Mooren's ulcer. Moreover, Lewallen and Courtright [15] stated that 29.6% of patients had trauma or surgery. Compared with the previous studies, fewer cases in our study were due to obvious trauma (7%) and previous ocular surgery (19%).We found that 45 out of 95 patients (47.3%) had a history of ocular surgery, corneal trauma, or infection, which was consistent with 41.7% reported by Kim et al. [4].

However, in a prospective study in India conducted by Sharma et al. [16], none of the eyes with Mooren's ulcer had a history of trauma or surgery as the inciting factors. In our study, there was no significant correlation between the recurrence of Mooren's ulcer and a history of ocular surgery or ocular trauma, whereas the significant correlation was found between the recurrence of Mooren's ulcer and a history of corneal infection. The possible reason that the proportion of ocular trauma in our study was lower than that in the previous reports might be the increasing awareness of protecting eyes in dangerous work environments in recent years.

In our research, the eyes in the recurrent group had a greater corneal perforation rate (31%) than that in the nonrecurrent group (7%). The survival analysis indicated that the existence of ocular infection and corneal perforation can induce recurrence of Mooren's ulcer. These two factors can lead to early recurrence and increased severity of Mooren's ulcer. Likewise, Zegans and Srinivasan [10] confirmed a statistically significant association between hookworm infection and Mooren's ulcer formation in his prospective cohort study of 21 patients in South India.

Mooren's ulcer is the result of an autoimmune process involving cell-mediated and humoral components [17]. Cornea-associated antigen (Co-Ag) has been found in the sera of patients with Mooren's ulcer [18]. In Akpek et al.'s and Gottsch et al.'s [19, 20] studies, one Co-Ag might be a protein named calgranulin C which is involved in the immune response to parasitic infections and can be also found in the corneal stroma. Therefore, calgranulin C is potentially a key factor in the pathogenesis of Mooren's ulcer.

Our study found that corneal infection and corneal perforation were related to recurrence of Mooren's ulcer. These two factors might cause early recurrence. Our study is one of the first to explore the association between the corneal infection/perforation and the recurrence of Mooren's ulcer. The findings provide a new insight into what factors can be used to predict the prognosis of Mooren's ulcer and how to better manage the treatment of subjects with Mooren's ulcer to prevent recurrence. Moreover, our findings may help reveal potential mechanisms of recurrence.

The retrospective nature of our study and the small sample size might be limitations of our study, while the low incidence of Mooren's ulcer limits our capacity to carry out a large prospective study. A prospective study including a variety of races and regions would help us to further understand the clinical characteristics, risk factors, and prognosis of this relentless disease.

## Supplementary Material

Additional table The clinical condition of repeatedly recurrent patients.

## Figures and Tables

**Table 1:** Demographics of patients.

Variables	Total (*n* = 95)	Recurrent group (*n* = 42)	Nonrecurrent group (*n* = 53)	*χ* ^2^/*t* value	*p*
Gender (male)	58 (61%)	27 (64%)	31 (58%)	0.331	0.565
Age	50 ± 14	47 ± 14	52 ± 14	−1.586	0.116
*Urbanicity*				0.263	0.608
Urban	25 (26%)	11 (26%)	14 (26%)		
Rural	70 (74%)	31 (74%)	39 (74%)		
*Single/double eyes*				0.533	0.465
Single	90 (95%)	39 (93%)	51 (96%)		
Double	5 (5%)	3 (7%)	2 (4%)		

**Table 2:** Clinical characteristics of affected eyes.

Variables	Total (*n* = 100)	Recurrent group (*n* = 45)	Nonrecurrent group (*n* = 55)	*χ* ^2^/*t* value	*p*
*Eye category*				0.206	0.650
Right	44 (44%)	19 (42%)	25 (46%)		
Left	56 (56%)	26 (58%)	30 (54%)		
*Corneal history*					
Corneal surgery				3.335	0.068
Yes	19 (19%)	12 (27%)	7 (13%)		
No	81 (81%)	33 (73%)	48 (87%)		
Corneal infection				**5.474**	**0.019**
Yes	19 (19%)	13 (29%)	6 (11%)		
No	81 (81%)	32 (71%)	49 (89%)		
Ocular trauma				0.492	0.483
Yes	7 (7%)	4 (9%)	3 (6%)		
No	93 (93%)	41 (91%)	52 (94%)		
Corneal perforation				**9.900**	**0.002**
Yes	18 (18%)	14 (31%)	4 (7%)		
No	82 (82%)	31 (69%)	51 (93%)		
*Corneal neovascularization*				0.000	1.000
Yes	45 (45%)	20 (44%)	25 (46%)		
No	55 (55%)	25 (56%)	30 (54%)		
*Epithelial defect*				2.942	0.086
Yes	10 (10%)	7 (16%)	3 (6%)		
No	90 (90%)	38 (84%)	52 (94%)		
*Corneal loose suture*					
Yes		6 (13%)			
No		39 (87%)			
*Clock hours of ulcer involvement*				3.364	0.339
>3 clock hours	37 (37%)	13 (29%)	24 (44%)		
>3 clock hours and ≤6 clock hours	34 (34%)	18 (40%)	16 (29%)		
>6 clock hours and ≤9 clock hours	16 (16%)	9 (20%)	7 (13%)		
>9 clock hours and ≤12 clock hours	13 (13%)	5 (11%)	8 (14%)		

**Table 3:** Clinical treatment of affected eyes.

Variables	Total (*n* = 100)	Recurrent group (*n* = 45)	Nonrecurrent group (*n* = 55)	Fisher value	*p*
*Treatment*				4.889	0.420
Medicine	14 (14.0%)	3 (6.7%)	11 (20.0%)		
Surgery methods^∗^	86 (86.0%)	42 (93.3%)	44 (80.0%)		
Partial LKP	48 (48.0%)	24 (53.3%)	24 (43.7%)		
Total LKP	21 (21.0%)	10 (22.2%)	11 (20.0%)		
AMT	13 (13.0%)	7 (15.6%)	6 (10.9%)		
CF	4 (4.0%)	1 (2.2%)	3 (5.4%)		

^∗^LKP: lamellar keratoplasty; AMT: amniotic membrane transplantation; CF: conjunctival flap.

**Table 4:** Clinical outcomes of affected eyes.

Variables	Total (*n* = 100)	Recurrent group (*n* = 45)	Nonrecurrent group (*n* = 55)	*χ* ^2^/*t* value	*p*
Inpatient days	11 (8, 16)	12 (8, 16)	11 (8, 15)	−0.575	0.565
*Visual Acuities*					
Initial V/A^∗^				4.332	0.228
<0.01	19 (19)	10 (22)	9 (16)		
0.01~0.1	23 (23)	14 (31)	9 (16)		
0.1~0.4	36 (36)	13 (29)	23 (42)		
0.4~1	22 (22)	8 (18)	14 (26)		
Final V/A				3.271	0.352
<0.01	18 (18)	9 (20)	9 (17)		
0.01~0.1	22 (22)	13 (29)	9 (16)		
0.1~0.4	36 (36)	15 (33)	21 (38)		
0.4~1	24 (24)	8 (18)	16 (30)		

^∗^V/A: visual acuities.
