# Supplementary material for: Clinical Characteristics and Risk Factors of Recurrent Mooren's Ulcer
Source: J Ophthalmol. 2017 Jun 27;2017:8978527. doi: 10.1155/2017/8978527 (PMC5504946; doi:10.1155/2017/8978527)
Supplement: Supplementary file 1 — Additional table The clinical condition of repeatedly recurrent patients. [file 8978527.f1.docx]

**Additional Table**

Additional table The clinical condition of repeatedly recurrent patients

| ID | sex | Presentation times | age | Combined medical illness | Clock hours of corneal involvement | Recurrence-free interval(day) | Signs of presentation | Conditions of affected eyes | Surgical treatment |
| --- | --- | --- | --- | --- | --- | --- | --- | --- | --- |
| Patient1 | male | 3 | 56 | Chemical trauma of right eye | left，from 2 to 4 | . | Ulcer of corneal graft | NV | AMT |
|  |  |  |  |  | left，from 2 to 4 | 12 | Red, pain，decreased vision | Impending perforating | Total LKP |
|  |  |  |  |  | left，from 2 to 4 | 9 | Red, pain，decreased vision | Fungal hyphae was found by corneal scraping | Total LKP |
| Patient 2 | female | 3 | 47 | hemiplegia | right，from 2 to 6 | . | Injured by branches | Nasal ulcer | Partial LKP+CF |
|  |  |  |  |  | right，from 5 to 6 | 70 | Foreign body sensation and pain | Nasal ulcer，NV | Partial LKP+AMT |
|  |  |  |  |  | right，from 3 to 5 | 400 | Foreign body sensation, red and pain | Inferior ulcer | Partial LKP |
| Patient3 | male | 3 | 42 | healthy | left，from 3 to 11 | . | Red, pain，decreased vision | ulcer | Total LKP+AMT |
|  |  |  |  |  | left，from 3 to 8 | 16 | Epithelium deficiency of corneal graft | Epithelium deficiency of central corneal graft | medicine |
|  |  |  |  |  | left，from 3 t 0 5 | 7 | Epithelium deficiency of corneal graft | Epithelium deficiency of central corneal graft | medicine |
| Patient 4 | female | 3 | 18 | healthy | left，from 3 t 0 7 | . | Red, pain，decreased vision | NV | Partial LKP |
|  |  |  |  |  | Left, From 5 to 7 | 90 | Epithelium deficiency of cornea | epithelium deficiency of inferior cornea graft，NV | medicine |
|  |  |  |  |  | left，from 5to6 | 330 | Recurrent ulcer | Ulcer, NV | CF |
| Patient 5 | male | 3 | 55 | healthy | left，from 7 to 12 | . | Red, pain，decreased vision | NV，corneal graft tilt | Total LKP+AMT |
|  |  |  |  |  | left，from 7 to 12 | 25 | Epithelium deficiency of corneal graft | Epithelium deficiency of central corneal graft，NV，liquid between layers | AMT |
|  |  |  |  |  | right，from 8to 12 | 6years and 1 month | Foreign body sensation, red, pain and tearing | ulcer，NV | Partial LKP |
| Patient 6 | male | 3 | 29 | Corneal ulcer of affected eye | Right ,from5to 13 | . | Red, pain，decreased vision | Nasal graft tilt | Partial LKP |
|  |  |  |  |  | right，from 4 to 8 | 27 | Red ,pain | Nasal graft tilt | Partial LKP+AMT |
|  |  |  |  |  | right，from 4 to 8 | 138 | Red, pain，decreased vision | Ulcer of central corneal graft | Partial LKP+AMP+CF |
| Patient 7 | male | 8 | 60 | gastric ulcer | right，from 3 to 2 | . | Red, pain，decreased vision | Peripheral ulcer | Total LKP+AMT |
|  |  |  |  |  | right，full cycle | 25 | Foreign body sensation, | Peripheral ulcer | Total LKP+AMT |
|  |  |  |  |  | right，from 10 to 11 | 22 | Foreign body sensation, | Amniotic dissolution | AMT |
|  |  |  |  |  | right，from 10 to 13 | 75 |  | Corneal graft dissolution | AMT |
|  |  |  |  |  | right，from 5to 7 | 20 |  | ulcer | CF |
|  |  |  |  |  | right，full cycle | 23 |  | ulcer | CF+AMT |
|  |  |  |  |  | right，from 5 to 9 | 42 |  | ulcer | Partial LKP+AMT |
|  |  |  |  |  | left**，2** clock | 150 | Red | ulcer | Partial LKP+AMT |
| Patient 8 | female | 8 | 50 | hypertension | right，from 5 to 7 | . | Red and pain | ulcer | Partial LKP |
|  |  |  |  |  | right，from 4 to 8 | 570 | Foreign body sensation, pain | ulcer，NV | Partial LKP+AMT |
|  |  |  |  |  | right，from 2 to 6 | 600 | red | ulcer，NV | AMT |
|  |  |  |  |  | right，from 2 to 8 | 495 | Foreign body sensation, | ulcer | AMT |
|  |  |  |  |  | right，from 3 to 7 | 222 | Tearing, photophobia | ulcer | Partial LKP |
|  |  |  |  |  | right，8 clock | 130 | Red, photophobia | ulcer, NV | AMT |
|  |  |  |  |  | right，from 6 to 8 | 123 | Red, tearing and photophobia | ulcer, NV | medicine |
|  |  |  |  |  | right，from 6 to 8 | 48 | Red, pain，decreased vision | ulcer | CF |
| Patient 9 | male | 5 | 57 | healthy | left，from 7 to 10 | . | Red, pain，decreased vision | ulcer | Partial LKP |
|  |  |  |  |  | left，from 7 to 1 | 1095 | Red, pain and tearing | ulcer, NV | CF |
|  |  |  |  |  | left，from 7 to 10 | 50 | Foreign body sensation | ulcer，perforating | Partial LKP |
|  |  |  |  |  | right，7 clock | 70 | Foreign body sensation, tearing | ulcer, NV | medicine |
|  |  |  |  |  | right，from 5 to 10 | 50 | Foreign body sensation | ulcer | Partial LKP |

Note: LKP, Lamellar Keratoplasty; AMT, Amniotic membrane transplantation; CF, Conjunctival Flap; NV, new vessels.
