# Supplementary material for: Clinical Characteristics and Risk Factors of Recurrent Mooren's Ulcer
Source: J Ophthalmol. 2017 Jun 27;2017:8978527. doi: 10.1155/2017/8978527 (PMC5504946; doi:10.1155/2017/8978527)
Supplement: Supplementary file 2 [file 8978527.f2.pdf]

Additional Table

Additional table The clinical condition of repeatedly recurrent patients

ID	sex	Presentation times	age	Combined medical illness	Clock hours of corneal involvement	Recurrence-free interval(day)	Signs of presentation	Conditions of affected eyes	Surgical treatment
Patient1	male	3	56	Chemical trauma of right eye	left，from 2 to 4	.	Ulcer of corneal graft	NV	AMT
					left，from 2 to 4	12	Red, pain，decreased vision	Impending perforating	Total LKP
					left，from 2 to 4	9	Red, pain，decreased vision	Fungal hyphae was found by corneal scraping	Total LKP
Patient 2	female	3	47	hemiplegia	right，from 2 to 6	.	Injured by branches	Nasal ulcer	Partial LKP+CF
					right，from 5 to 6	70	Foreign body sensation and pain	Nasal ulcer，NV	Partial LKP+AMT
					right，from 3 to 5	400	Foreign body sensation, red and pain	Inferior ulcer	Partial LKP
Patient3	male	3	42	healthy	left，from 3 to 11	.	Red, pain，decreased vision	ulcer	Total LKP+AMT

					left，from 3 to 8	16	Epithelium deficiency of corneal graft	Epithelium deficiency of central corneal graft	medicine
					left，from 3 t 0 5	7	Epithelium deficiency of corneal graft	Epithelium deficiency of central corneal graft	medicine
Patient 4	female	3	18	healthy	left，from 3 t 0 7	.	Red, pain，decreased vision	NV	Partial LKP
					Left, From 5 to 7	90	Epithelium deficiency of cornea	epithelium deficiency of inferior cornea graft，NV	medicine
					left，from 5to6	330	Recurrent ulcer	Ulcer, NV	CF
Patient 5	male	3	55	healthy	left，from 7 to 12	.	Red, pain，decreased vision	NV，corneal graft tilt	Total LKP+AMT
					left，from 7 to 12	25	Epithelium deficiency of corneal graft	Epithelium deficiency of central corneal	AMT

								graft，NV，liquid between layers	
					right，from 8to 12	6years and 1 month	Foreign body sensation, red, pain and tearing	ulcer，NV	Partial LKP
Patient 6	male	3	29	Corneal ulcer of affected eye	Right ,from5to 13	.	Red, pain，decreased vision	Nasal graft tilt	Partial LKP
					right，from 4 to 8	27	Red ,pain	Nasal graft tilt	Partial LKP+AMT
					right，from 4 to 8	138	Red, pain，decreased vision	Ulcer of central corneal graft	Partial LKP+AMP+CF
Patient 7	male	8	60	gastric ulcer	right，from 3 to 2	.	Red, pain，decreased vision	Peripheral ulcer	Total LKP+AMT
					right，full cycle	25	Foreign body sensation,	Peripheral ulcer	Total LKP+AMT
					right，from 10 to 11	22	Foreign body sensation,	Amniotic dissolution	AMT
					right，from 10 to 13	75		Corneal graft	AMT

								dissolution	
					right，from 5to 7	20		ulcer	CF
					right，full cycle	23		ulcer	CF+AMT
					right，from 5 to 9	42		ulcer	Partial LKP+AMT
					left，**2** clock	150	Red	ulcer	Partial LKP+AMT
Patient 8	female	8	50	hypertension	right，from 5 to 7	.	Red and pain	ulcer	Partial LKP
					right，from 4 to 8	570	Foreign body sensation, pain	ulcer，NV	Partial LKP+AMT
					right，from 2 to 6	600	red	ulcer，NV	AMT
					right，from 2 to 8	495	Foreign body sensation,	ulcer	AMT
					right，from 3 to 7	222	Tearing, photophobia	ulcer	Partial LKP
					right，8 clock	130	Red, photophobia	ulcer, NV	AMT
					right，from 6 to 8	123	Red, tearing and photophobia	ulcer, NV	medicine
					right，from 6 to 8	48	Red, pain，decreased vision	ulcer	CF
Patient 9	male	5	57	healthy	left，from 7 to 10	.	Red, pain，decreased vision	ulcer	Partial LKP
					left，from 7 to 1	1095	Red, pain and tearing	ulcer, NV	CF
					left，from 7 to 10	50	Foreign body sensation	ulcer，perforating	Partial LKP

	right，7 clock	70	Foreign body sensation, tearing	ulcer, NV	medicine
	right，from 5 to 10	50	Foreign body sensation	ulcer	Partial LKP

Note: LKP, Lamellar Keratoplasty; AMT, Amniotic membrane transplantation; CF, Conjunctival Flap; NV, new vessels.
